# Predictive impact of the inflammation‐based indices in colorectal cancer patients with adjuvant chemotherapy

**DOI:** 10.1002/cam4.1542

**Published:** 2018-05-15

**Authors:** Yong Tao, Lei Ding, Guan Gen Yang, Jian Ming Qiu, Dong Wang, Hongtao Wang, Chao Fu

**Affiliations:** ^1^ Zhejiang Chinese Medicine University Affiliated No.3 Hangzhou Hospital Hangzhou China; ^2^ The Third People’s Hospital of Hangzhou Hangzhou China

**Keywords:** adjuvant chemotherapy, colorectal cancer, neutrophil‐to‐lymphocyte ratio, platelet‐to‐lymphocyte ratio

## Abstract

Increasing evidences reported that cancer‐triggered inflammation was associated with survival prognosis from colorectal cancer (CRC). However, the comprehensive effects of inflammatory‐based coNLR‐PLR that combines neutrophil‐to‐lymphocyte ratio (NLR) and platelet‐to‐lymphocyte ratio (PLR) rarely remain to be determined during chemotherapy. We retrospectively analyzed clinical data and baseline laboratory parameters from 153 colorectal cancer patients who underwent palliative adjuvant chemotherapy between January 2009 to January 2012. Receiver operating characteristic (ROC) curves and linear regression analyzed the predictive ability of NLR, and PLR for calculating the score of coNLR‐PLR. Overall survival (OS) and recurrence‐free survival (RFS) rates were estimated using the Kaplan‐Meier method and analyzed by the Cox proportional hazards model in univariate and multivariate analysis. The optimal cut‐off value of NLR and PLR was 2.24 and 186 by the ROC analysis. Kaplan‐Meier method showed that patients with high coNLR‐PLR score was associated with poorer OS and RFS (all *P* < .05). In univariate and multivariate analysis, it obtained that the coNLR‐PLR severed as a strong independent prognostic factor for OS and RFS (all *P* < .05). These results highlight that coNLR‐PLR index severed as a strong predictor of prognosis biomarker in CRC patients receiving adjuvant chemotherapy. Furthermore, its assessment could contribute to accurately predicting prognosis after chemotherapy in clinical practice.

## 1 INTRODUCTION

Recently, colorectal cancer (CRC) is regarded as a type of cancer associated with chronic inflammation and adjuvant chemotherapy was initially applied to the treatment of inoperable locally advanced tumors.^1^ Many randomized trials have confirmed that adjuvant chemotherapy has proven to benefit patients with CRC for several decades.^2^, ^3^ Despite notable improvements in the treatment of chemotherapy, prognostic outcome still remain poor in 5‐year survival.^4^ Therefore, it is essential to pay much attention exploring the pivotal molecular elements participating in tumor chemotherapy and determining effective prognostic biomarkers that are evaluated for chemotherapy efficacy. Generally, these predictive factors come from clinicopathological characteristics of tumor, such as stage, type, grade etc which have been widely utilized as biomarkers for predicting the prognosis of postchemotherapy.^5^ However, the increasing challenge of tumor management showed that it was difficult to predict the heterogeneous prognosis seen with similarly clinicopathological tumors including CRC. Hence, it is of urgent need to identify potential biomarkers that can predict associations between the survival prognosis and adjuvant chemotherapy.

It is well known that systemic inflammatory response always plays a vital role as a leading cause of neoplastic process, and it was actively engaged in genesis and propagation of various cancers,including CRC.^6^, ^7^ Increasing evidences suggest that systemic inflammation can be reflected by parameters of peripheral blood including white blood cells, neutrophils, lymphocytes, as well as platelets. At the time of prognosis, the neutrophil‐to‐lymphocyte ratio (NLR) and the platelet‐to‐lymphocyte ratio (PLR) have been confirmed to be the systemic inflammatory response indicators for many malignancies such as biliary tract cancer,^8^ gastric cancer,^9^ and lung cancer.^10^ To our knowledge, NLR and/or PLR have been also previously linked to survival or response to treatment in CRC.^11^ Those results indicated that both NLR and PLR could participate in inflammatory response to cancer biology and have guiding role for the current clinical treatment. However, results of some published studies reported that there is a larger controversy in the consistency of these 2 inflammation‐based indicators.^12^, ^13^

In order to comprehensively explore the predictive value, we hypothesized that a novel prognostic score system combined NLR and PLR would reflect more accurately exhibit behaviors related to the prognosis of CRC patients. For this aim, we focused on the coNLR‐PLR score which could be examined the potential role in improving predictive capacity of established prognostic nomograms before chemotherapy.

## 2 METHODS AND MATERIALS

The study was approved by the ethics committee of Zhejiang Chinese Medicine University Affiliated No. 3 Hangzhou Hospital. Written informed consent was obtained from all patients, and was performed in accordance with the ethical standards of Declaration of Helsinki and its later amendments.

### 2.1 Patient and controls selection

We retrospectively reviewed altogether the records of 153 CRC patients treated with adjuvant chemotherapy in the Department of Colorectal Surgery at Zhejiang Chinese Medicine University Affiliated No. 3 Hangzhou Hospital from January 2009 to January 2012. Patients were enrolled under the following inclusion criteria: (1) all patients underwent curative surgery for CRC, whose expected survival prognosis was not less than 5 years; (2) no patients with infection, hematological diseases, hyperpyrexia, renal dysfunction; (3) no drug use, including NSAIDs; (4) vascular disorder or inflammation‐related diseases; (4) no previous chemotherapy, radiotherapy, or other targeted therapy.

### 2.2 Adjuvant chemotherapy protocol

All patients were treated with the adjuvant chemotherapy regimen of mFOLFOX6 or XELOX according to National Comprehensive Cancer Network (NCNN) guidelines. mFOLFOX6 regimen: oxaliplatin (OXA) 85 mg/m^2^, levofolinate calcium (l‐LV) 200 mg/m^2^, bolus 5‐fluorouracil (5‐FU) 400 mg/m^2^, all on day 1; infusion 5‐FU 2400 mg/m^2^ on days 1‐3. Once every 2 weeks, for 6 cycles. XELOX regimen: oxaliplatin (OXA) 130 mg/m^2^ on day 1; capecitabine (1000 mg/m^2^) on days 1‐14. Once every 3 weeks, for 8 cycles. The duration of patients who had received surgery was within 1 month.

### 2.3 Peripheral NLR and PLR score method

Hematological parameters were detected before the first cycle of chemotherapy by Sysmex XT‐1800i Automated Hematology System (Hangzhou, China). For the calculation of the NLR and PLR, NLR was defined as the absolute neutrophil count divided by the absolute lymphocyte count and PLR was also conducted by the same way. According to the ROC curves and significant correlation with each other, we defined the scores of NLR as 1 or 0 when patients had a high or a low NLR value before receiving adjuvant chemotherapy. Similarly, the PLR scores were 1 or 0 when patients had, respectively, a high or a low PLR. The combined score (coNLR‐PLR) was defined as follows: patients with both high NLR and high PLR were assigned a score of 2, and patients scoring high for only one parameter, or low for both, were assigned a score of 1 or 0, respectively.

### 2.4 Data collection and follow up

Medical records were collected to find data on each patient’s age, gender, clinicopathological characteristics (such as location, size, histological type, TNM stage, invasion, lymph node), and laboratory data (such as NLR and PLR). All patients were followed regularly by letters and telephone interviews every 3‐6 months until death or 5 years. The recurrence‐free survival (RFS) was defined as the time from the first day of palliative chemotherapy to the disease progression or recurrence. The overall survival (OS) time was defined as the time from first day of palliative chemotherapy to death by any cause or to the last follow‐up. Of all, the follow‐up duration was for 5 years and ended after January 1, 2017.

### 2.5 Statistical analysis

As predictive markers for OS, the optimal cut‐off values for NLR and PLR was determined by the receiver‐operating characteristic (ROC). Linear regression was performed to evaluate the association between NLR and PLR. The association between clinical‐pathological characteristics and coNLR‐PLR score were compared by the Chi‐square test or Fisher’s exact probability test. For the analysis of prognosis, OS and PFS were calculated using the Kaplan‐Meier method and the differences were compared using the log‐rank test. The Cox proportional hazards model with 95% confidence interval was used for the univariate and multivariate analysis to assess the effect of patient characteristics and other significant prognostic factors. A *P* value of .05 or less was considered statistically significant. All analysis were performed using SPSS software for Windows (version 11.5).

## 3 RESULT

### 3.1 Patient characteristics and comparison in healthy controls

Detailed clinicopathological information of 153 patients is shown in Table 1. Of all CRC patients, the mean age was 62.31 ± 11.59 years old and male‐to‐female ratio was 81 vs 72. 61 patients suffered from colon cancer, while 92 suffered from rectal cancer. Evaluation of TNM stages revealed that the pathological diagnoses were 51 patients of stage II and 102 patients of stage III‐IV. Moreover, all patients received first adjuvant chemotherapy treatment. Among them, there were more than 27 patients with larger tumor size (≥5 cm) respectively. Histological CRC grade contained 99 patients of G1/G2 and 54 patients of G3/G4. In terms of cancer invasion, higher grade (T3/T4) had 87 patients and the other grade (T1/T2) had 66. The lymph node with negative tumor (N0) was 79 while the positive had 74 patients. Compared with the basis line parameters of the healthy controls, CRC patients had higher serum CEA, CRP levels, baseline neutrophil count, platelet counts, but lower baseline lymphocyte compared to that of controls (all *P* < .05). It indicated that changes in the distribution of the WBC subsets were reflected in higher values of NLR and PLR in the patient group.

**Table 1:** Demographic information of colorectal cancer (CRC) patients and healthy controls

Characteristics	CRC patients	Healthy controls	*P* value
Age (y)	62.31 ± 11.59	63.51 ± 11.92	.594
Male/Female	81/72	79/74	.819
Serum CEA (ng/mL)^2^	6.30 ± 1.67	2.79 ± 1.34	<.001
Serum CRP (mg/L)^5^	17.22 ± 2.94	2.61 ± 1.75	<.001
Baseline WBC (×109/L)^4^	6.61 ± 2.43	4.68 ± 0.75	.194
Baseline neutrophil count (×109/L)	4.18 ± 1.75	2.64 ± 0.91	.004
Baseline lymphocyte count (×109/L)	1.87 ± 0.45	2.49 ± 0.33	.002
Baseline Platelet count (×109/L)	244.30 ± 31.53	183.00 ± 51.00	.001
Location			
Colon	61 (39.87%)		
Rectal	92 (60.13%)		
Tumor size (cm)			
<5	63 (41.18%)		
≥5	90 (58.82%)		
Cancer stage			
II	51 (33.33%)		
III‐IV	102 (66.67%)		
Cancer grade			
G1/G2	99 (64.71%)		
G3/G4	54 (35.29%)		
Tumor invasion			
T1/T2	66 (43.13%)		
T3/T4	87 (56.82%)		
Lymph node			
N0	79 (51.63%)		
N+	74 (48.37%)		

### 3.2 Calculation of NLR, PLR and coNLR‐PLR score

**Figure 1:**
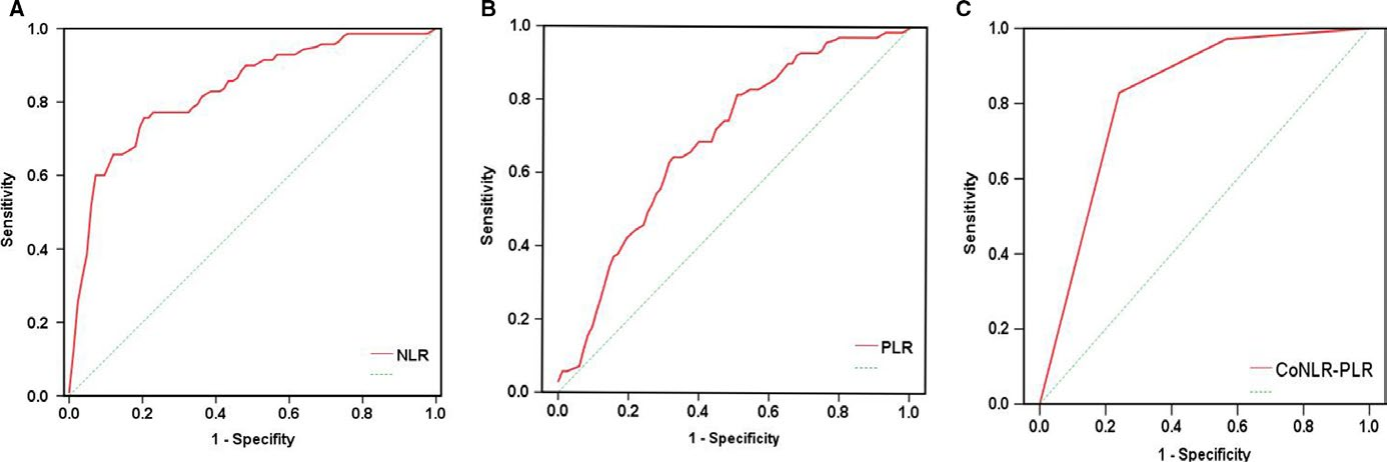
Receiver operating characteristic curve analyses. A, For neutrophil‐to‐lymphocyte ratio (NLR), it is indicated with an area under the curve (AUC) = 0.804 (95% CI 0.765‐0.896, *P* < .001), a sensitivity of 78.62%, a specificity of 62.34%. B, For platelet‐to‐lymphocyte ratio (PLR), it is indicated with an AUC = 0.616 (95%CI 0.607‐0.774, *P* < .001), a sensitivity of 67.94%, a specificity of 59.26%. C, For coNLR‐PLR, it is indicated with an AUC = 0.831 (95%CI 0.752‐0.889, *P* < .001), a sensitivity of 72.91%, a specificity of 80.71%

**Figure 2:**
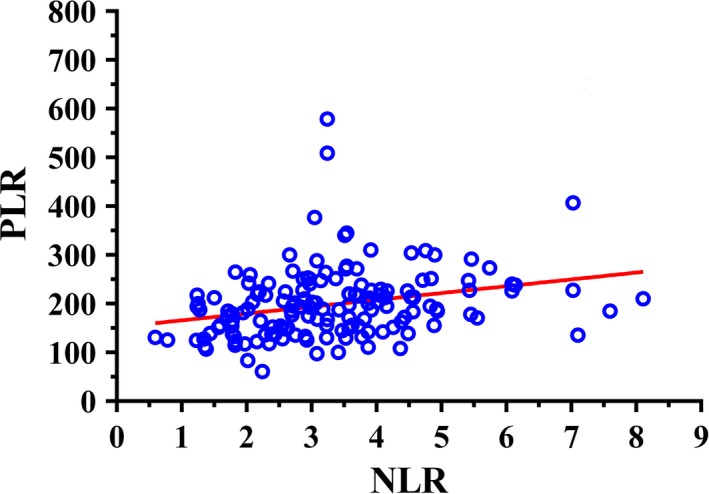
Linear regression of neutrophil‐to‐lymphocyte ratio (NLR) and platelet‐to‐lymphocyte ratio (PLR). NLR and PLR were associated with each other (*R* ^2^ = 0.5368, *P* < .001)

**Figure 3:**
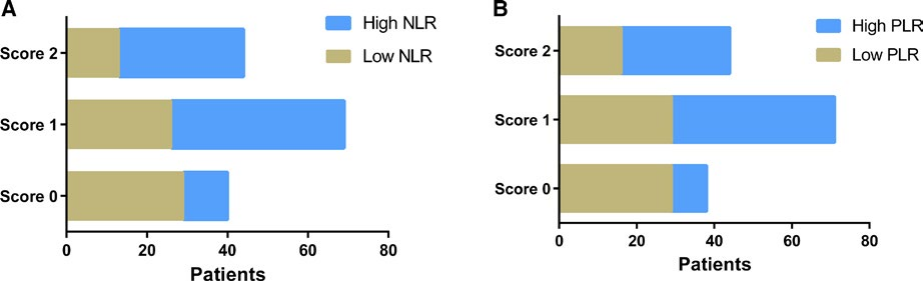
The distribution between scores and CRC patients. For A, patients with high/low NLR in score groups. For B, patients with high/low PLR in score groups.

ROC curve could calculate the sensitivity and specificity levels of NLR and PLR as predictors of OS. It concluded that the optimal cut‐off value of NLR and PLR were calculated 2.24 and 186 by the AUC with the You‐den index in Figure 1. Linear regression indicated the significant association between NLR and PLR (*P* < .001) in Figure 2. Moreover, the combined score (coNLR‐PLR) was defined as follows: patients with both high NLR and high PLR were assigned a score of 2, and patients scoring high for only one parameter, or low for both, were assigned a score of 1 or 0, respectively. Figure 3 showed the distribution between scores and CRC patients. It also indicated that only parameter of NLR scores were more closely associated with total coNLR‐PLR scores than the PLR scores in this present study.

### 3.3 Relationships of NLR, PLR, and coNLR‐PLR in clinicopathological characteristics

Table 2 shows the distribution of the clinicopathological characteristics of the studied patients grouped according to NLR and PLR. Patients with high cancer stage, poor cancer grade, sever tumor invasion included significantly more elevated NLR and PLR (all *P* < .05). On the contrary, there was no difference in such basis line as age, gender, tumor location and size, and lymph node. Table 3 shows the associations between the characteristics and the 3 groups of patients separated according to the coNLR‐PLR score. Significant intergroup differences were found for cancer stage, tumor invasion, and lymph node metastasis (all *P* < .05). Moreover, NLR and PLR, which served as either categorical data or measurement data, also showed significant differences among the 3 groups (all *P* < .001).

**Table 2:** Relationships between clinical characteristics and NLR or PLR

Characteristics	TotalN = 153	NLR		*P* value	PLR		*P* value
		≥2.24	<2.24		≥186	<186	
Age (y)							
<60	64	33	31	.326	28	36	.098
≥60	89	52	37		51	38	
Sex							
Female	72	39	33	.744	42	30	.118
Male	81	46	35		37	44	
Location							
Colon	61	37	24	.301	31	30	.780
Rectal	92	48	44		48	44	
Tumor size (cm)							
<5	63	31	32	.186	37	26	.115
≥5	90	54	36		42	48	
Cancer stage							
II	51	14	37	<.001	16	35	<.001
III‐IV	102	71	31		63	39	
Cancer grade							
G1/G2	99	49	50	.041	43	56	.006
G3/G4	54	36	18		36	18	
Tumor invasion							
T1/T2	66	29	37	.012	25	41	.026
T3/T4	87	56	31		54	33	
Lymph node							
N0	79	39	40	.111	36	43	.121
N+	74	46	28		43	31	

NLR, neutrophil‐to‐lymphocyte ratio; PLR, platelet‐to‐lymphocyte ratio.

**Table 3:** Relationships between clinical characteristics and coNLR‐PLR

Characteristics	TotalN = 153	coNLR‐PLR			*P* value
		Score 0	Score 1	Score 2	
Age (y)					
<60	64	17	32	15	.467
≥60	89	21	39	29	
Sex					
Female	72	17	36	19	.695
Male	81	21	35	25	
Location					
Colon	61	14	26	21	.451
Rectal	92	24	45	23	
Tumor size (cm)					
<5	63	16	23	24	.063
≥5	90	22	48	20	
Cancer stage					
II	51	22	18	10	.001
III‐IV	102	16	53	34	
Cancer grade					
G1/G2	99	21	51	27	.194
G3/G4	54	17	20	17	
Tumor invasion					
T1/T2	66	24	23	19	.008
T3/T4	87	14	48	25	
Lymph node					
N0	79	27	36	16	.007
N+	74	11	35	28	
NLR					
<2.24	68	29	26	13	<.001
≥2.24	85	9	45	31	
PLR					
<186	74	24	34	16	<.001
≥186	79	14	37	28	

CoNLR‐PLR, combined neutrophil‐to‐lymphocyte ratio and platelet‐to‐lymphocyte ratio.

### 3.4 Survival outcome

**Figure 4:**
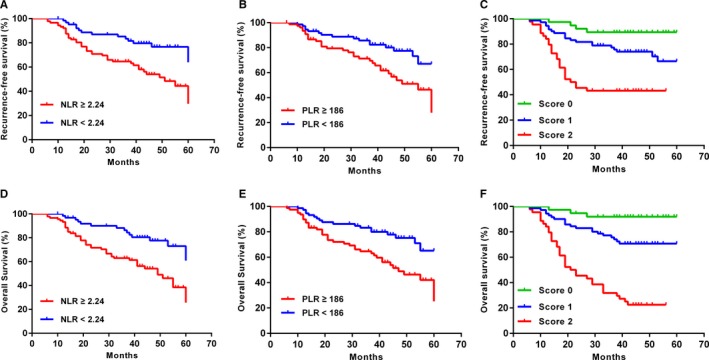
RFS and OS curves grouped by neutrophil‐to‐lymphocyte ratio (NLR), platelet‐to‐lymphocyte ratio (PLR), and coNLR‐PLR. A, B, C, Patients with NLR ≥ 2.24, PLR ≥ 186 and high coNLR‐PLR scores had inferior RFS(log rank *P* < .05 for all). D, E, F, Patients with NLR ≥ 2.24, PLR ≥ 186, and high coNLR‐PLR scores had inferior OS (log rank *P* < .05 for all)

During the follow‐up period, 51 patients (33.3%) developed tumor recurrence. Among those, 18 showed local recurrence and 31 developed metastasis. 61 patients (39.87%) died, 4 from cardiovascular and cerebrovascular events, 1 in a traffic accident, 1 from chemotherapeutic toxicity, 51 from tumor recurrence, and the other 4 due to unknown reasons. Tumors recurred in 3 out of 38 patients (7.89%) with a coNLR‐PLR score of 0, 20 out of 71 patients (28.17%) with a coNLR‐PLR score of 1, and 28 out of 44 patients (63.64%) with a coNLR‐PLR score of 2 (log‐rank *P* < .001). Death occurred in 3 patients (5.9%) with a coNLR‐PLR score of 0, 20 patients (27.7%) with a coNLR‐PLR score of 1, and 34 patients (73.0%) with a coNLR‐PLR score of 2 (log‐rank *P* < .001). With regard to RFS and OS, we compared survival prognosis in potential factors including NLR, PLR and coNLR‐PLR score by Kaplan‐Meier curve and log‐rank test in Figure 4. The results indicated that patients with elevated NLR, PLR, and high coNLR‐PLR score had in a significantly poorer prognosis for RFS and OS (all *P* < .05).

### 3.5 Independent prognostic factors for RFS and OS

For analysis of all prognostic factors, Tables 4 and 5 show the results of univariate and multivariate analysis of various parameters with RFS and OS evaluated in this study. Univariate analysis indicated that patients with high cancer stage (III‐IV), elevated NLR (≥2.24) and PLR (≥186), as well as higher coNLR‐PLR score were obviously associated with worse RFS and OS (all *P* < .05). However, tumor invasion was only associated obviously with OS, while cancer grade and lymph node were only for RFS, respectively. Moreover, factors with *P* < .05 in univariate analysis, were conducted in the COX model for further multivariate analysis. It suggests that both high cancer stage, NLR, and coNLR‐PLR score were related to inferior RFS and OS (all *P* < .05). However, PLR was only significantly associated with worse RFS while NLR was for OS only. Thus, the coNLR‐PLR score system can effectively classify patients into 3 independent groups.

**Table 4:** Univariate and multivariate analysis of OS using the Cox proportional hazard model

Variables	Univariate analysis			Multivariate analysis		
	HR	95% CI	*P* value	HR	95% CI	*P* value
Age						
<60	Referent			‐	‐	‐
≥60	0.949	0.567‐1.587	.842			
Gender						
Male	Referent			‐	‐	‐
Female	1.048	0.511‐2.146	.899			
Location						
Rectal	Referent			‐	‐	‐
Colon	0.645	0.378‐1.075	.092			
Tumor size						
<5	Referent			‐	‐	‐
≥5	0.459	0.274‐0.768	.103			
Cancer stage						
II	Referent					
III‐IV	2.981	1.632‐5.445	<.001	2.049	1.099‐ 3.968	<.001
Cancer grade						
G1/G2	Referent			‐	‐	‐
G3/G4	1.326	0.781‐2.249	.296			
Tumor invasion						
T1/T2	Referent					
T3/T4	2.972	1.502‐5.879	.002	1.228	0.749‐ 2.011	.415
Lymph node						
N0	Referent			‐	‐	‐
N+	1.449	0.855‐2.456	.169			
NLR						
<2.24	Referent					
≥2.24	2.319	1.360‐3.955	.004	1.794	1.112‐ 2.893	.027
PLR						
<186	Referent					
≥186	1.375	0.472‐1.973	.012	1.522	0.812‐ 2.871	.189
CoNLR‐PLR						
0	Referent					
1	3.438	2.356‐4.124	<.001	1.453	1.002‐ 2.716	.182
2	5.540	3.275‐9.372	<.001	1.987	1.188‐ 3.180	.010

CoNLR‐PLR, combined neutrophil‐to‐lymphocyte and platelet‐to‐lymphocyte ratio; NLR, neutrophil‐to‐lymphocyte ratio; PLR, platelet‐to‐lymphocyte ratio.

**Table 5:** Univariate and multivariate analysis of RFS using the Cox proportional hazard model

Variables	Univariate analysis			Multivariate analysis		
	HR	95% CI	*P* value	HR	95% CI	*P* value
Age						
<60	Referent			‐	‐	‐
≥60	1.045	0.561‐1.946	.890			
Gender						
Male	Referent			‐	‐	‐
Female	0.941	0.378‐2.436	.897			
Location						
Rectal	Referent			‐	‐	‐
Colon	0.597	0.323‐1.101	.099			
Tumor size						
<5	Referent			‐	‐	‐
≥5	0.487	0.263‐0.902	.022			
Cancer stage						
II	Referent			Referent		
III‐IV	2.983	1.318‐6.751	.009	1.941	1.255‐ 3.011	.003
Cancer grade						
G1/G2	Referent			Referent		
G3/G4	2.376	1.605‐4.100	.011	1.672	0.759‐ 3.681	.202
Tumor invasion						
T1/T2	Referent			‐	‐	‐
T3/T4	1.615	0.867‐3.009	.131			
Lymph node						
N0	Referent			Referent		
N+	3.529	1.766‐7.052	.001	1.501	0.601‐ 3.749	.385
NRL						
<2.24	Referent			Referent		
≥2.24	1.490	0.789‐2.814	.019	1.394	0.558‐ 3.481	.476
PLR						
<186	Referent			Referent		
≥186	2.054	1.109‐3.802	.022	1.685	1.077‐ 2.672	.020
CoNLR‐PLR						
0	Referent					
1	1.250	1.052‐1.484	.011	1.344	1.028‐ 1.766	.031
2	3.529	1.766‐7.052	.001	2.492	1.182‐ 5.242	.016

## 4 DISCUSSION

Although the adjuvant chemotherapy of colorectal cancer have greatly improved several decades, survival outcomes still remain a poor prognosis and leads most common cause of cancer‐related death.^14^ For the need of better survival, it is vital to find some accurate and sensitive indicators for predicting prognosis.

To our knowledge, tumor and systemic inflammation have close relationships between each other.^15^ It confirmed that inflammatory response plays an essential role in the progression of tumor microenvironment and some changes of inflammatory cells might be as a predictor for prognosis. Various studies have indicated that changes of immune cellular components in peripheral venous blood could reflect tumor inflammation status for predicting survival prognosis.^16^ There is a growing interpretation of the relationship between inflammation and tumor, resulting in the establishment of novel biomarkers of cancer to evaluate the prognostic significance.^17^ It is reported that neutrophils reflect the systematic inflammation status and accelerate the extracellular matrix remodel, which stimulate the tumor cell proliferation, migration, and metastasis through its enzymatic actions, such as the release of reactive oxygen species (ROS), nitric oxide (NO), and proteases.^18^ Moreover, neutrophil could activate inflammation to promote tumor growth by proangiogenic factors and growth factors.^19^ Another reason is the lymphocyte infiltration response to the tumor. Increased lymphocytic reactions have been connected with a better prognosis in chemotherapy.^20^, ^21^

Also to the findings mentioned above, PLR is another systemic inflammation biomarker. It is well known that platelet and lymphocyte counts were reported to be associated with prognosis in CRC chemotherapy as circulating biomarkers for inflammation, immune response, and coagulation status.^22^ It is conformed that CRC patients who received oxaliplatin‐based combination chemotherapy had worse disease control than those with a high PLR.^23^ The underlying mechanisms responsible for the role of PLR in tumor chemotherapy have not yet been elucidated, but recent experimental and clinical data may provide several potential explanations. A growing body of evidence reported that high PLR can activate the invasiveness of tumor cells by enhancing the formation of tumor stoma and supporting the stable adhesion of tumor cells to the endothelium.^24^ In addition, platelets could secrete cellular growth factors such as platelet‐derived growth factor, vascular endothelial growth factor, transforming growth factor beta, platelet factor 4, and then stimulate tumor angiogenesis and growth.^25^

Due to these inflammatory indicators with intrinsic correlations, recent studies have reported that prechemotherapy NLR and PLR are useful factors for predicting tumor response after chemotherapy in various cancer.^26^ However, there is a great deal of risk and bias in only one index or detection. For more comprehensive assessment on prognosis, we used the coNLR‐PLR score system that combined these 2 inflammation‐based indices to analyze OS and RFS in this present study, which could more accurately reflect the values of NLR and PLR before chemotherapy. Instead of being stratified into only 3 scores (0, 1, and 2) as with Glasgow prognostic score,^27^ the coNLR‐PLR score system is calculated by the cut‐off value of their ROC curves. As far as known, this is the first study exploring the association of the coNLR‐PLR index with prognosis in CRC patients with chemotherapy. It was demonstrated that such index could serve effectively as a stronger, independent prognostic factor and higher score group is closely in consistent with poor prognosis for OS and RFS. The coNLR‐PLR system combines two inflammation‐related indices, NLR and PLR, so it may reflect the systemic inflammatory response (SIR) status more comprehensively, and is arguably a superior predictor of prognosis. Besides coNLR‐PLR, cancer stage was also identified as a robust prognostic indicator in the univariate and multivariate analysis, which was consistent with the results of previous studies.^28^, ^29^ Compared with these prognostic indicators, these inflammatory biomarkers have advantages of being simple, easily available, economical, objective, and reproducible and could be measured at the beginning of chemotherapy treatment and monitored throughout the entire therapy periods.^30^ In addition, the complementary combination of coNLR‐PLR could form a more complex model, which may provide firm prognostic information for clinicians.

Our study has some limitations that deserve to be mentioned. First, it is a retrospective investigation, so it exists as a potential bias in the selection of patients. Second, we did not evaluate the adverse reactions from chemotherapy, which might have affected the quality of patient life and survival. Finally, whether such inflammatory indicators could be incorporated into the stratification system of cancer patients to address individual treatment needs has to be clarified in future prospective studies.

## 5 CONCLUSION

In summary, our study demonstrated that the novel indicator of coNLR‐PLR system is independently correlated with the survival prognosis, suggesting the important roles of peripheral blood cells in bridging the cancer‐host interaction during the different steps of adjuvant chemotherapy. Due to the retrospective design of the current study, further prospective studies are required to validate our findings.

## CONFLICT OF INTEREST

All authors have no conflict of interest to declare.
